# Prostatic Massage

**Published:** 1905-02

**Authors:** W. L. Champion

**Affiliations:** Atlanta, Ga.; Genito-Urinary Surgeon, The Halcyon-Retreat


### PROSTATIC MASSAGE.*

By W. L. CHAMPION, M.D.,
ATLANTA, GA.
Genito-Urinary Surgeon, The Halcyon-Retreat.

While the title of this paper is "Prostatic Massage," the involvement of the seminal vesicles and glands of the urethra will also be discussed. The methods suggested as beneficial and curative do not apply to prostatic hypertrophy, but involvement of the glandular structures by extension of urethral infection. While advances have not been made as rapidly in genito-urinary work recently as in other lines, the acknowledged fact that urethral infection extends to the adjacent glandular structures, and a field has been opened whereby relief from these conditions can be obtained, by massage properly performed, is an advance in keeping with other pathological conditions. In this short paper it is not necessary to discuss the anatomy and physiology of the prostate, but only to state "the prostate is a sexual organ, partly glandular, partly muscular, lying in front of the bladder about the prostatic urethra. The prostate is the sexual heart. It has nothing to do with urination, and is quite passive during this act. But towards the sexual function it acts as a muscle, a sensory organ, and a gland."

More can be learned in regard to the size, diseased conditions of the prostate by rectal examinations, than all other means combined.

There are various positions in which patients are placed to examine and massage the prostate. I have the patients stand with elbows on seat of a chair, legs straight and feet separated. Use pressure over bladder with left hand, and massage prostate with forefingers of right hand. The use of the rubber finger cot makes the treatment less objectionable to the patient and more cleanly to the surgeon. The fingers should be passed up to the base of the prostate and pressure made as the finger is brought towards the apex. Firm pressure should be made over the entire gland, noting the impression made upon the patient. If he becomes fainty limit

*Read before Atlanta Society of Medicine, January 19, 1905.

the examination or massage to a few seconds and make a more thorough massage at the next treatment, when the patient will tolerate more pressure upon the gland on account of a less sensitive condition due directly to the previous massage. In highly nervous individuals, or those in which the gland is very sensitive, massage every fourth or fifth day is as often as advisable. In some cases I use massage only once a week, while in others with urine cloudy, dull heavy sensation in gland with frequent desire to urinate, I massage daily if the patient is made more comfortable by such treatment. The amount of pressure made upon the gland, the intervals between the treatments and the length of each seance must be determined by results obtained in each individual case. When a prostate is massaged, instruct the patient to have some urine in the bladder so that after the treatment the urine can be voided in a glass so as to note what secretion is compressed out of the gland. Always irrigate the bladder after massage with an antiseptic solution so as to cleanse it of any septic material that may be present. Stones or concretions in the gland should be felt for, the condition of the vesicles, and any nodules or other abnormalities noted. The vesicles are above the prostate and lie on the outer side of each vas deferens. Stripping of the vesicles is necessary to remove gonorrheal infection, and the genito-urinary surgeon is called upon to determine if the patient is sterile, by obtaining the semen to note if spermatozoa are present and alive. By massage of the prostate we compress out the infected secretion and stimulate a better circulation in the gland and adjacent diseased structures. Where there is a spasmodic condition of the neck of the bladder and deep urethra, massage relieves the sensitive condition and spasm so that the patient is made more comfortable and irrigation of the bladder more easily accomplished. To illustrate that sensation is blunted it is noticeable that a patient can not empty the bladder immediately after prostatic massage. Lydston has well said that "sexual neurasthemia embracing bowel inactivity, melancholia, hypochondreasis, and that seldom recognized but frequent condition, male hysteria, are among the results of chronic prostatic disease, which are frequently relievable by proper and systematic massage."

A frequently obscure condition, but one due to a local irritation, abnormal nocturnal emissions and premature ejaculation are frequently benefited or relieved by massage. I recall a case I had a few months ago of a boy sixteen years of age who had wet the bed every night since his birth, and a single massage caused him to pass a week without wetting the bed.

While prostatic massage is indicated in deep-seated infection due usually to gonorrhea, I mention these conditions to show the correcting influence it may have on other irregularities. "Clap shreds" seen in the urine of chronic cases that remain after seemingly all treatment has been exhausted, will many times disappear after a systematic course of massage. Massage of the urethra on a steel or soft sound will give results in cases where other means have failed. While I do not claim prostatic massage as a cure-all, it is a recognized procedure that will relieve heretofore obscure diseased conditions of the genito-urinary apparatus.

*Prudential Building.*

